# Psychological Impact of the COVID-19 Outbreak on Nurses in China: A Nationwide Survey During the Outbreak

**DOI:** 10.3389/fpsyt.2020.598712

**Published:** 2020-12-11

**Authors:** Yan Liu, Youlin Long, Yifan Cheng, Qiong Guo, Liu Yang, Yifei Lin, Yu Cao, Lei Ye, Yan Jiang, Ka Li, Kun Tian, Xiaoming A, Cheng Sun, Fang Zhang, Xiaoxia Song, Ga Liao, Jin Huang, Liang Du

**Affiliations:** ^1^Emergency Department of West China Hospital, Sichuan University/West China School of Nursing, Sichuan University, Chengdu, China; ^2^Institute of Disaster Medicine, Sichuan University, Chengdu, China; ^3^West China School of Medicine, Sichuan University, Chengdu, China; ^4^Department of Urology, West China Hospital, Sichuan University, Chengdu, China; ^5^Department of Emergency, West China Hospital, Sichuan University, Chengdu, China; ^6^Nursing Department, West China Hospital, Sichuan University, Chengdu, China; ^7^West China School of Nursing, Sichuan University, Chengdu, China; ^8^West China Hospital, Sichuan University, Chengdu, China; ^9^Neuro-Intensive Care Unit, Affiliated Hospital of Chifeng University, Chifeng, China; ^10^Emergency Intensive Care Unit, The First People's Hospital of Yunnan Province/The Affiliated Hospital of Kunming University of Science and Technology, Kunming, China; ^11^Department of Cardiology, Guangzhou First People's Hospital, South China University of Technology, Guangzhou, China; ^12^Department of Rheumatology and Immunology, Union Hospital Affiliated With Tongji Medical College, Huazhong University of Science and Technology, Wuhan, China; ^13^Department of Emergency, China-Japan Friendship Hospital, Beijing, China; ^14^State Key Laboratory of Oral Diseases, National Clinical Research Center for Oral Diseases, West China Hospital of Stomatology, Sichuan University, Chengdu, China; ^15^West China Medical Publishers, West China Hospital, Sichuan University, Chengdu, China

**Keywords:** COVID-19, nurses, mental health, infectious disease, pandemic (COVID-19)

## Abstract

**Background:** The COVID-19 pandemic is a major public health issue and challenge to health professionals. In similar epidemics, nurses experienced more distress than other providers.

**Methods:** We surveyed both on-duty nurses caring for infected patients and second-line nurses caring for uninfected patients from Hubei and other provinces throughout China.

**Results:** We received completed surveys from 1,364 nurses from 22 provinces: 658 front-line and 706 second-line nurses. The median (IQR) GHQ-28 score of all nurses was 17 (IQR 11–24). The overall incidence of mild-to-moderate distress (GHQ score > 5) was 28%; that for severe distress (GHQ score > 11) was 6%. The incidence of mild-to-moderate distress in the second-line nurses was higher than that in the front-line nurses (31 vs. 25%; OR, 0.74; 95 CI, 0.58–0.94). Living alone (OR, 0.62; 95% CI, 0.44–0.86) and feeling supported (OR, 0.82, 95% CI, 0.74–0.90) independently predicted lower anxiety.

**Conclusions:** During the COVID-19 pandemic, the psychological problems of all nurses were generally serious. The interviewed second-line nurses face more serious issues than the front-line nurses.

## Introduction

The 2019 outbreak of the new coronavirus disease (COVID-19) in China is an epidemic threat and major public health issue (1). The World Health Organization (WHO) declared this outbreak a public health emergency of international concern on January 30, 2020 (2). As of March 4, 2020, COVID-19 had been spread to all provinces and regions of China and to 75 other countries. In some regions, the cumulative number of COVID-19 cases may continue to rise (3). This indicates that the epidemic may continue to worsen in some countries. The Chinese Center for Disease Control and Prevention (CDC) reported on February 17 estimated that more than 3,000 healthcare workers were infected with COVID-19 in China. Studies of Severe Acute Respiratory Syndrome (SARS) (4, 5), Middle Eastern Respiratory Syndrome (MERS-CoV) (6), and COVID-19 (7, 8) have reported that many healthcare workers including nurses caring for patients during these epidemics had distress, anxiety, and other mental health problems (9). Chen et al.'s (4) study showed that the SARS catastrophe affected the stress levels in the emergency department, and Khalid et al. (6) confirmed that the MERS-CoV outbreak was a distressing time for the medical staff. For example, during the SARS outbreak, many healthcare workers were stigmatized and shunned in their neighborhoods as a result of their jobs (10–12). Treating SARS patients led to mental health problems among many emergency department staff, with nurses experiencing the most stress, followed by doctors and healthcare assistants (13). Health workers in many countries involved in the treatment of COVID-19 have been under considerable pressure since the COVID-19 outbreak (14–17). Most of the medical workers fighting COVID-19 are nurses. As of February 9, 2020, an estimated 19,800 health care professionals, including 14,000 nurses, from across China have provided assistance to hospitals in Hubei province, especially Wuhan City (18). Nurses generally have long-term and close contact with suspected and confirmed COVID-19 patients. Under these conditions, the coping ability of many nurses begins to decline, a change often neglected by the healthcare system (6). Consequently, the mental health of nurses working with patients infected with COVID-19 need to be monitored and maintained through an epidemic. However, we have not found any article that focuses specifically on nurses' mental health during the COVID-19 outbreak. Samui et al.'s (19) findings suggested that COVID-19 would persist for a long time. We sought to describe the mental health of nurses in China during the COVID-19 outbreak.

## Methods

### Study Design and Participants

Between February 11 and 18, 2020, during the COVID-19 outbreak, we conducted an online survey of nurses who were working during the COVID-19 outbreak in China, whether or not they were treating patients with COVID-19. The survey was approved by the Biomedical Research Ethics Committee, West China Hospital of Sichuan University.

**Figure 1:**
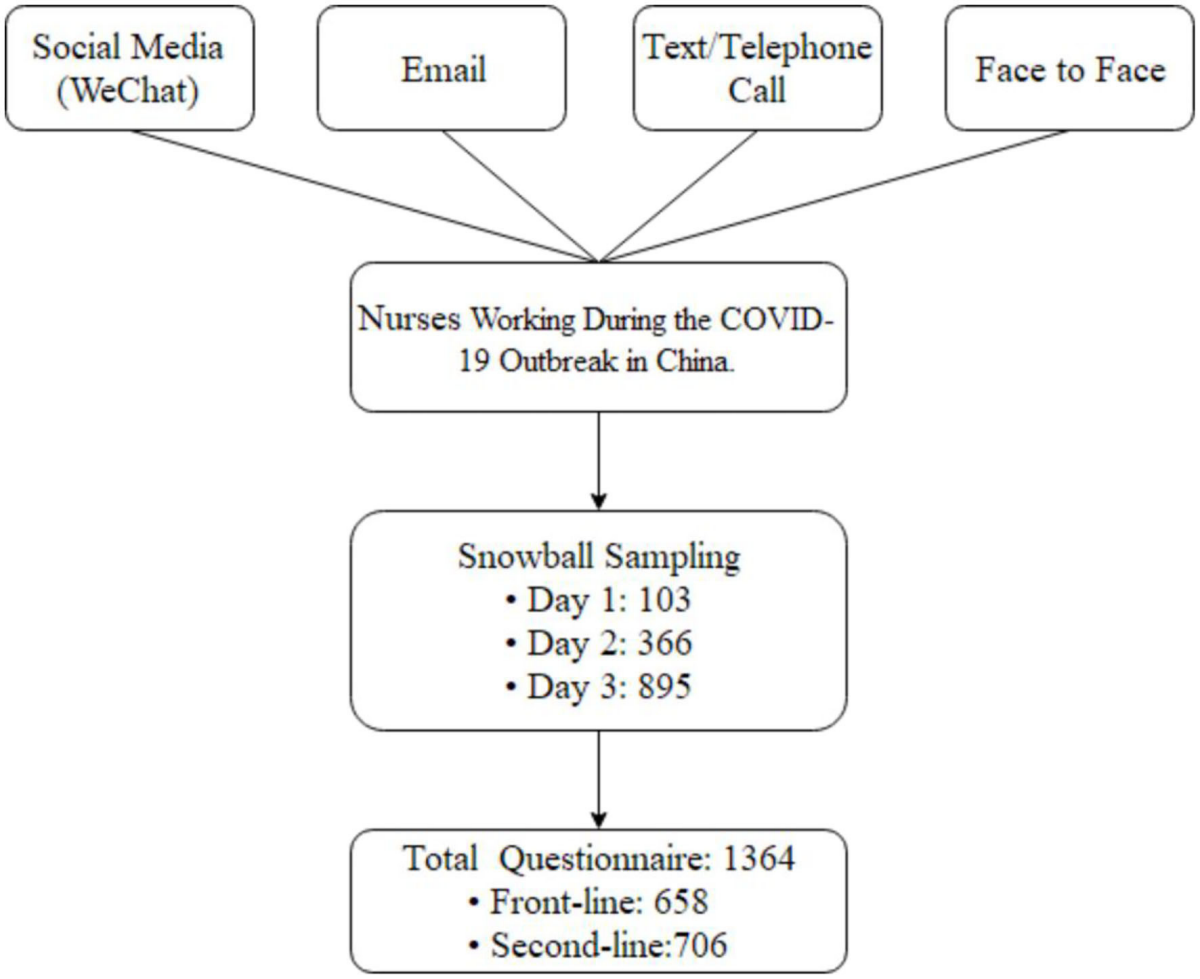
The flow chart of questionnaires distribution and nurses selection. WeChat is a communication software that can forward files.

We selected some nurses who we knew according to the inclusion criteria, and then we used snowball sampling in which the initial nurses recommended the survey to other nurses who in turn recommended the survey to more nurses (Figure 1). A message about the study and a guarantee of anonymity were sent to all responding nurses. We distributed a questionnaire by SO JUMP (a professional online questionnaire platform) to all invited nurses. The questionnaire was administered directly to the nurses who volunteered via WeChat (a kind of communication software that can forward files), or the questionnaires were given to the nurses by the volunteers (most of them were medical workers) via WeChat. All potential participants were informed about research purposes and good confidentiality. The questionnaire was anonymous and all data were kept confidential by a special researcher. Nurses were told that their participation was voluntary and that they could stop any time. Each received the survey only after verbal informed consent was obtained. To avoid duplicated submissions, the questionnaires were set for only one chance by WeChat. To ensure that respondents were part of the target population, the questionnaire QR code was sent only to those who met inclusion criteria.

The questionnaire could not be submitted until all questions had been answered. To eliminate questionnaires not filled carefully, questionnaires returned within 150 s were excluded from analysis to eliminate ineligible questionnaires.

### The Questionnaire

The questionnaire was administered on-line and in Chinese, the native language of all respondents. It consisted of 86 questions in six parts: demographic information, sources of information and degree of concern about the epidemic, perceived sufficiency of information, anxiety-related behavior and perceived support, degree of distress, and coping strategies (Table 1). Degree of distress was measured with the validated Chinese version of the General Health Questionnaire-28 (GHQ-28), a 28-item self-report instrument developed to screen for the inability to carry out normal functions and to detect the appearance of new and distressing phenomena. The instrument measures four dimensions: depression, anxiety, social impairment, and somatic symptoms. The minimum clinically important difference and the minimal detectable change have not been determined (20). We ran a predictive test on 10 nurses. The result showed that it took 5 min on average to complete the questionnaire and 3 min at a minimum.

**Table 1:** Characteristics of the questionnaire used to assess psychological distress.

**Part**	**Dimension**	**Questions**
**1**	Demographic characteristics	12 questions on age, sex, educational background, professional title, occupation, department, marital status, having children, and living alone)
**2**	Sources of information and degree of concern about the epidemic	10 questions, 5 dichotomous items, and 5 scored on a 9-point Likert scale (1 low; 9 high) on degree of concern and reasons for the concern
**3**	Perceived sufficiency of information	8 questions, 7 scored on a 9-point Likert scale (1 low; 9 high) and 1 on a 5-point scale on the degree of information desired about the pandemic (1 low; 5 high)
**4**	Anxiety-related behavior and perceived support	15 questions, 4 on worry, 11 dichotomous items about the adequacy of various forms of support, 3 of intended behaviors., and 1 about work satisfaction scored on a 9-point Likert scale (1 highly probable; 9 impossible)
**5**	Participants' level of distress	The Chinese version of the General Health Questionnaire-28 (GHQ-28), a 28-question measure of emotional distress in medical settings. Scores range from zero (no distress) to 84 (maximum distress)
**6**	Participants' coping strategies	13 questions on the frequency of coping behaviors. Participants endorsed how often they used a particular coping strategy scored on a 4-point Likert scale (0 never; 3 very often)

### Statistical Methods

Data are summarized with means and standard deviations or medians and interquartile ranges and were analyzed with SPSS software (version 18.0; SPSS Inc., Chicago, Illinois). Alpha was set at 0.05, and all tests were two-tailed. Total GHQ scores can range from 0 to 28 and were calculated with the dichotomous scoring procedure (0–0–1–1). Scores between 5 and 10 defined mild-to-moderate distress, and scores of 11 or above defined severe distress (21). Scores on the four subscales (depression, anxiety, social impairment, and somatic symptoms) were summed to calculate the total score. Chi-square analyses, Wilcoxon rank-sum tests, and two sample two-tailed *t*-test assessed differences in basic characteristics, concerns, worries, degree of worry, perceived sufficiency of information, GHQ-28 scores, and social support between front- and second-line nurses. We also reported odds ratios (OR) and 95% confidence intervals for dichotomous data, as well as mean differences and 95% confidence intervals for continuous data when comparing data from front-line and second-line nurses. Chi-square tests, two-sample Wilcoxon rank-sum tests, and Spearman's rank correlation analysis were used to assess associations between intended behaviors and worries and degree of worry about the COVID-19 pandemic. Unadjusted and multiple logistic regression analyses were conducted to explore factors associated with worries and distress (total GHQ scores above and below a score of 5), including demographic variables, participation in treating patients with COVID-19, social support, and coping strategies. Missing data were imputed with the sample mean for the variable.

## Results

### Sample Description

By February 16, 2020, 1,364 questionnaires had been returned, all of which yielded valid data. There was no missing data. The 658 front-line nurses and 706 second-line nurses represented 22 provinces and regions in China (Figure 1). The distribution had no obvious regional concentration. Median age was 30.0 (IQR 28–34) years. About one-fifth were men (*n* = 292, 21%). Front-line nurses had significantly more years of education than second-line nurses and significantly more years of service (medians of 8 and 7 years, respectively). A third of all nurses lived alone, with significantly more front-line nurses than second-line nurses reporting living alone (Table 2).

**Table 2:** Demographic characteristics of All 1,364 Chinese nurses.

**Characteristic**	**Total *N* = 1,364**	**Front-line nurses *n* = 658**	**Second-line nurses *n* = 706**	***P*-value**
**Age, median (IQR)**, years	30 (27-34)	31 (2-34)	30 (26-35)	0.051
**Women**, *n* (%)	1,072 (79%)	507 (77%)	565 (80%)	0.18
**Education background**, *n* (%)				0.02
PhD	6 (0.4%)	4 (0.6%)	2 (0.3%)	
Master	40 (3%)	17 (3%)	23 (3%)	
Bachelor	1,032 (76%)	519 (79%)	513 (73%)	
College degree and others	286 (21%)	118 (18%)	168 (24%)	
**Professional Title**, *n* (%)				0.27
Advanced	75 (5%)	27 (4%)	48 (7%)	
Medium-grade	386 (28%)	209 (32%)	177 (25%)	
Primary	903 (66%)	422 (64%)	481 (68%)	
**Years of service**, median (IQR), years	8 (4-12)	8 (5-12)	7 (3–12)	0.04
**Manager**, *n* (%)	268 (20%)	125 (19%)	143 (20%)	0.56
**Marital status**, *n* (%)				0.95
Married	868 (64%)	420 (64%)	448 (63%)	
Unmarried	463 (34%)	223 (34%)	240 (34%)	
Divorced	33 (2%)	15 (2%)	18 (3%)	
**Living with a child**, *n* (%)	799 (59%)	383 (58%)	416 (59%)	0.79
**Lives alone**, *n* (%)	447/917 (33%)	233 (35%)	214/492 (30%)	0.045

*Front-line nurses provided care for patients with the COVID-19 infection or suspected COVID-19 infection; second-line nurses did not*.

### Degree of Distress

Eighty-eight percent of the nurses worried that COVID-19 might pose a pandemic threat, which contributed to their distress. The median anxiety score was about seven of nine for all nurses. Their most common concerns were the risk of infection in family members or relatives (92%), the risk of infection (89%), the risk of being isolated from family and society (77%), and the impact of their career planning (31%). Notably, the percentage of second-line nurses reporting distress was higher than that of the front-line nurses for all of these concerns. Similarly, median severity scores for becoming infected and being treated for the infection were significantly higher in second-line nurses than in front-line nurses (Table 3). Unadjusted logistic regression analysis showed that spinsterhood (OR = 0.704, *P* = 0.04), divorce (OR = 0.366, *P* = 0.02), living alone (OR = 0.605, OR = 0.003), and total support scores (OR = 0.814, *P* < 0.001) were significantly associated with less anxiety about the pandemic, but in the multivariable analysis, only living alone (OR = 0.616, *P* = 0.004) and social support (OR = 0.817, *P* < 0.001) were independently related to anxiety (Table 4).

**Table 3:** Sources of distress reported by 1,364 Chinese nurses during the COVID-19 pandemic.

**Source of distress**	**Front-line nurses**	**Second-line nurses**	***P*-value**
	***n* = 658**	***n* = 706**	
	***n* (%)**	***n* (%)**	
**I worry about the COVID-19 pandemic**, ***n*** **(%)**	568 (86%)	631 (89%)	0.08
**Degree of worry [median (IQR)]** 1, low; 9, high	7 (5–9%)	7 (5–8%)	0.21
**I mostly worry about**
The disease's danger, ***n*** **(%)**	571 (86.8%)	640 (90.7%)	0.02
The risk that family and relatives will be infected, ***n*** **(%)**	594 (90.3%)	666 (94.3%)	0.005
Isolation from family or social environment, ***n*** **(%)**	488 (74.2%)	557 (78.9%)	0.04
Damage to my future career development, ***n*** **(%)**	174 (26.4%)	252 (35.7%)	< 0.001
**Perceived risk for being infected by the COVID-19 [median (IQR)]** 1, very low; 9, high	6 (4–8)	6 (5–7)	0.72
**Being infected with the COVID-19 would have major consequences on my health [median (IQR)]** 1, low; 9, high	6 (5–8)	7 (5–9)	0.001
**The infection is difficult to treat [median (IQR)]** 1, low; 9, high	5 (3–7)	5 (4–7)	<0.001
**My department is well prepared for the COVID-19 pandemic [median (IQR)]** 1, low; 9, high	7.5 (6–9)	7 (5–8)	<0.001

**Table 4:** Analysis of influencing factors of that nurses are worried about the COVID-19 pandemic.

**Variable**	**Univariate analysis (Logistic regression, Enter)**		
	**Beta**	***P***	**OR (95% CI)**
**Age, years**	0.018	0.19	1.018 (0.991–1.046)
**Sex**	0.296	0.12	1.345 (0.925–1.956)
**Education background**			
PhD vs. College degree and others	−0.361	0.75	0.697 (0.079-6.143)
Master vs. College degree and others	−0.024	0.96	0.976 (0.359–2.657)
Bachelor vs. College degree and others	0.021	0.92	1.021 (0.684–1.524)
**Professional title**			
Advanced vs. primary	0.22	0.57	1.247 (0.584–2.662)
Medium-grade vs. primary	0.253	0.19	1.288 (0.880–1.884)
**Service years**	0.017	0.18	1.017 (0.992–1.042)
**Whether a manager**			
(Yes/No) (Yes = 1/No = 0)	0.302	0.18	1.352 (0.869–2.103)
**Marital status**			
Spinsterhood vs. Married	−0.35	0.04	0.704 (0.502–0.989)
Divorced vs. married	−1.005	0.02	0.366 (0.160–0.835)
**Whether have a child** (Yes/No) (Yes = 0/No = 1)	−0.298	0.07	0.742 (0.535–1.029)
**Whether living alone** (Yes/No) (Yes = 1/No = 0)	−0.503	0.003	0.605 (0.434–0.843)
**Total support score**	−0.206	<0.001	0.814 (0.736–0.899)

Outcomes of multivariate analysis showed that only living alone and social support were independently related to anxiety. [B, P, OR (95%CI)]: Whether living alone^*^ [−0.484, 0.004, 0.616 (0.441–0.860)]; Total support score^*^ [−0.202, <0.001, 0.817 (0.739–0.903)].

### Perceived Adequacy of Epidemic-Related Information

The front-line nurses' median scores estimating information for treatment and prevention were significantly higher. The clarity of the information provided by their departments about infection and prevalence of COVID-19 was scored 9 of 9 (IQR, 7–9), which was higher than the second-line nurses' 8 (IQR, 7–9; *P* = 0.02). First- and second-line nurses were in desperate need of health-related information. The median score for “your demand on health-related information” was 5 (IQR, 5–5; Table 5).

**Table 5:** Perceived sufficiency of information about the COVID-19 pandemic and general health information needs.

**Type of information**	**Total**	**Front-line**	**Second-line**	***P*-value**
	**Median (IQR)**	**Median (IQR)**	**Median (IQR)**	
**I believe that I have heard sufficient information about** (1, strongly disagree; 9, strongly agree)				
COVID-19 symptoms	8 (7-9)	8 (7-9)	8 (7-9)	0.67
COVID-19 prognosis	7 (6-8)	7 (6-8)	7 (5-8)	0.11
COVID-19 treatment	7 (5-8)	7 (6-8)	7 (5-8)	<0.001
COVID-19 infection route	8 (7-9)	8 (7-9)	8 (7-9)	0.79
COVID-19 preventive measures	8 (7-9)	8 (7-9)	8 (7-9)	0.04
**I believe that my department provided clear information about the COVID-19 influenza pandemic** (1, strongly disagree; 9, strongly agree)	9 (7-9)	9 (7-9)	8 (7-9)	0.26
**Overall, the information I have heard about COVID-19 has been clear** (1, strongly disagree; 9, strongly agree; five items Cronbach's alpha, 0.89)	8 (7-9)	8 (7-9)	8 (7-9)	0.02
**General health-information needs for a disease I might contract** (1, I prefer having no more information than needed; 5, I prefer as much information as possible)	5 (5)	5 (5)	5 (5)	0.89

### Anxiety and Social Support

Thirty-eight percent of nurses reported feeling isolated from family and friends as a result of high-risk exposure. The proportion of nurses feeling isolated was significantly higher in front-line nurses than second-line nurses (42 vs. 34%, OR, 1.45; 95% CI, 1.16–1.80). More than three-quarters of all nurses reported that the high risk of exposure at work limited their socialization. Only 20 (1.5%) nurses said that they might ask for leave from work for fear of infection. The top three sources of sufficient support were team spirit among colleagues (97%), support from friends and family (93%), and new work arrangements and clear guidelines for infection control (90%). The item “Had insurance and was compensated if infected at work” had the lowest sufficient support (74%). The proportion of nurses reporting sufficient support from all sources was higher in front-line than in second-line nurses and significantly higher for six sources. Total support points were significantly lower in second- than in front-line nurses (8.7 vs. 8.2; Table 6). Anxiety was significantly associated with “Feeling they were isolated from family and friends because of a high risk of infection” (*P* = 0.005) and to having to limit socialization because of this risk as well (*P* < 0.001; Table 7).

**Table 6:** Presence of anxiety-producing behavior and social support among 1,364 Chinese nurses during the COVID-19 pandemic.

**Behavior**	**All nurses**	**Front-line nurses**	**Second-line nurses**	***P-*value**
**Isolation** (I feel that my family members and friends avoid contacts with me, because I work in a “high-risk” environment), ***n*** **(%)**	517 (38%)	279 (42%)	238 (34%)	0.001
**Restriction of Social Contacts** (I have restricted my social contacts because my work environment is considered “dangerous”), ***n*** **(%)**	1,043 (77%)	509 (77%)	534 (76%)	0.46
**Intended Work Avoidance** (Lately I have been so concerned about the COVID-19 influenza that I would take a leave to avoid going to work), ***n*** **(%)**	20 (1.5%)	10 (1.5%)	10 (1.4%)	0.87
**Sense of Duty** (In an emergency situation due to the COVID-19 pandemic, how possible would it be to avoid your duties? (1, highly possible; 9, not at all possible), **Median (IQR)**	9 (8,9)	9 (8,9)	9 (8,9)	0.001
**Support items** (inadequate vs. adequate), ***n*** **(%)**				
Support from relatives	1257 (92%)	611 (93%)	646 (92%)	0.35
Appreciation from the community	1166 (86%)	587 (89%)	579 (82%)	<0.001
Protective facilities and temporary residential arrangements	1069 (78%)	542 (82%)	527 (75%)	0.001
Insurance and compensation	1011 (74%)	520 (79%)	491 (69%)	<0.001
Sense of coherence and team spirit	1322 (97%)	639 (97%)	683 (97%)	0.69
Gratitude from patients and their relatives	1135 (83%)	561 (85%)	574 (81%)	0.051
Clear infection control guideline	1231 (90%)	607 (92%)	624 (88%)	0.02
Frontline staff feedback reaching administrators	1174 (86%)	581 (88%)	593 (84%)	0.02
Counseling and psychological support from employer	1093 (80%)	547 (83%)	546 (77%)	0.007
Expressing opinions through staff unions or mass media	1090 (80%)	540 (82%)	550 (78%)	0.055
**Other behaviors**, ***n*** **(%)**	1044 (77%)	518 (79%)	526 (75%)	0.07
**Total support score, Median (IQR)**	10 (8,10)	10 (8,10)	9 (7-10)	<0.001

**Table 7:** Association between “Worry about the COVID-19 pandemic” and anxiety-producing behaviors among 1,364 Chinese nurses during the COVID-19 pandemic.

**Anxiety-Producing Behavior**		**Worry about the COVID-19 pandemic**		***P-value***
		**Yes, *n* (%)**	**No, *n* (%)**	
**Isolation** (I feel that my family members and friends avoid contacts with me, because I work in a “high-risk” environment)	Yes	471 (39%)	46 (28%)	0.005
**Restriction of Social Contacts** (I have restricted my social contacts because my work environment is considered “dangerous”)	Yes	942 (79%)	101 (61%)	<0.001
**Intended Work Avoidance** (Lately I have been so concerned about the COVID-19 that I would take a leave to avoid going to work)	Yes	19 (1.6%)	1 (0.6%)	0.53
**Sense of Duty** (In an emergency situation due to the COVID-19 pandemic, how possible would it be to avoid your duties?) (1, highly possible; 9, not at all possible)	Mean (IQR)	9 (8–9)	9 (8–9)	0.19

### Psychological Distress

The incidence of mild-to-moderate distress (GHQ scores > 5) in all nurses was 28%, and the incidence in second-line nurses was higher than that in front-line nurses (31 vs. 25%; OR = 1.35, 95% CI, 1.06–1.71, *P* = 0.01). In addition, the incidence of severe distress (GHQ scores > 11) in all nurses was 6% but did not differ significantly between front- and second-line nurses (Table 8). Univariate logistic regression analysis showed that nurses who lived alone (OR, 0.72; 95% CI, 0.56–0.94), had closer first-line contact with COVID-19 infected patients (OR, 0.72; 95% CI, 0.54–0.94), and had higher support scores (OR, 0.77; 95% CI, 0.73–0.81) had lower incidence of mild-to-moderate distress. However, multivariable regression analysis showed that only higher support scores were independently associated with lower distress (OR, 0.77; 95% CI, 0.72–0.82; Table 9).

**Table 8:** Scores on the Chinese version of the general health questionnaire-28 for identifying minor psychiatric disorders completed by 1,364 Chinese nurses during the COVID-19 pandemic.

**Dimension**	**All nurses**	**Front-line nurses**	**Second-line nurses**	***P*-value**
**Total score, median (IQR)**	17 (11–24)	16 (10–23)	18 (11-24)	0.07
**Mild distress** (score >5), ***n*** **(%)**	378 (28%)	162 (25%)	216 (31%)	0.01
**Severe distress** (score >11), ***n*** **(%)**	75 (5.5%)	35 (5.3%)	40 (5.7%)	0.78

*Scores range from zero (no distress) to 84 (maximum distress)*.

**Table 9:** Characteristics associated with psychological distress among 1,364 Chinese nurses during the COVID-19 pandemic.

**Characteristic**	**Univariate analysis (Logistic regression,Enter)**		
	**B**	***P***	**OR (95%CI)**
**Age, years**	0.009	0.33	1.009 (0.991–1.028)
**Sex**	0.109	0.47	1.115 (0.831–1.495)
**Education background**
PhD vs. College degree and others	0.359	0.68	1.432 (0.257–7.983)
Master vs. College degree and others	0.433	0.23	1.543 (0.765–3.111)
Bachelor vs. College degree and others	0.103	0.50	1.109 (0.824–1.493)
**Professional Title**			
Advanced vs. Primary	0.055	0.84	1.056 (0.625–1.786)
Medium-grade vs. Primary	0.13	0.34	1.138 (0.875–1.482)
**Years of experience**	0.011	0.17	1.011 (0.995–1.028)
**Management position**	0.108	0.47	1.114 (0.830–1.495)
**Marital status**
Unmarried vs. married	−0.253	0.054	0.777 (0.601–1.004)
Divorced vs. married	−0.446	0.30	0.640 (0.274–1.493)
**Has a child (yes** **=** **0/no** **=** **1)**	−0.115	0.35	0.892 (0.700–1.136)
**Living alone (yes** **=** **1/no** **=** **0)**	−0.323	0.02	0.724 (0.558–.940)
**Whether to treat COVID-19 patients directly**			
Less contact with the COVID-19 patients vs. no	–0.241	0.15	0.786 (0.565–1.093)
Frequent contact with the COVID-19 patients vs. no	−0.335	0.02	0.716 (0.543–0.943)
**Support from relatives**	−1.035	<0.001	0.355 (0.238–0.530)
**Appreciation from the community**	−1.132	<0.001	0.322 (0.237–0.439)
**Protective facilities and temporary residential arrangements**	−0.94	<0.001	0.391 (0.298–0.512)
**Insurance and compensation**	−1.035	<0.001	0.355 (0.275–0.460)
**Sense of coherence and team spirit**	−1.499	<0.001	0.223 (0.118–0.421)
**Gratitude from patients and their relatives**	−0.826	<0.001	0.438 (0.326–0.588)
**Clear infection control guideline**	−1.307	<0.001	0.271 (0.188–0.390)
**Frontline staff feedback reaching administrators**	−1.095	<0.001	0.334 (0.244–0.458)
**Counseling and psychological support from employer**	−1.045	<0.001	0.352 (0.267–0.464)
**Expressing opinions through staff unions or mass media**	−1.001	<0.001	0.368 (0.279–0.484)
**Others**	−0.75	<0.001	0.472 (0.362–0.616)
**Total support score**	−0.267	<0.001	0.766 (0.727–0.807)
**Total score of stress coping strategies**	−0.009	0.83	0.991 (0.912–1.077)

*Only a low total support score was associated with distress on multivariable analysis*.

## Discussion

On February 13, Hubei province announced 14,840 new confirmed cases of COVID-19 infection, a sharp rise from only a few days before. Sarkar et al.s' (22) study shows that isolation can effectively reduce the number of COVID-19 infections, and that quarantine, isolation, and prevention measures play a vital role in the progress of the epidemic. Therefore, a large number of medical workers are needed for epidemic prevention and control. This first severe wave of the COVID-19 pandemic outbreak led to an acute shortage of nurses. More than 20,000 medical workers from across the country are now coping with COVID-19; three-quarters of them are nurses, and of these, nearly 80% are women. Despite the fact that they regarded COVID-19 as a horrible danger, they continued to treat their patients. Activities to prevent and control coronavirus pneumonia in China are ongoing, which continues to put medical workers under great pressure. In the H1N1 and Ebola outbreaks, nurses were the most vulnerable health care workers (23, 24). Protecting the mental health of nurses is thus important for controlling the epidemic and for their own long-term health (25). Nurses have the most direct contact with COVID-19 patients and also provide direct medical interventions (26). We found that front-line nurses were more highly educated and had more experience than did the second-line nurses. Nurses who preferred going to the front line had higher seniority and education and were more likely to live alone. As a result, the front-line nurses differed from the second-line nurses because they had more experience with infectious diseases, a finding similar to that in Liu et al.s'(27) study of a Chinese medical team working in the Sierra Leone aid mission treating Ebola patients. Nurses in relation to the COVID-19 outbreak were stressed and worried that their friends and relatives might be infected. Both the front-line nurses and the second-line nurses were very worried about the COVID-19 outbreak. This was probably the main reason nurses felt stressed. The stress may change the nurses' career plans. The government and their organizations had provided separate accommodation for the front-line nurses. But the second-line nurses are stressed more, so some of them chose to live apart from their family or to stay at the hotel after work at their own expense. The second-line nurses thought that their departments were ill-prepared for this new infectious disease. They were more worried about their health and thought the disease was difficult to control. The most frequent concern among 93% of nurses was that their families and friends would become infected, perhaps because their elder relatives might have chronic conditions, which is associated with more severe infections (28, 29). In addition, the pandemic began during the Spring Festival, the most important traditional festival in China, when people return to their hometowns. Many infections were asymptomatic. The second-line nurses were more likely to take care of them. If these patients were infected but asymptomatic, the second-line nurses were at high risk of infection. So, more of them worried about infecting their families and friends. In our survey, more than three-quarters of both first- and second-line nurses reduced their social interactions. The reason might be they did not know whether the patients they treated were infected, and most did not have adequate protective equipment (30). Lack of protective equipment increases the risk of infection and distress of front-line nurses (27, 31). Despite their own lack of protective equipment, some second-line nurses preferred that this equipment go to front-line nurses, who needed them more. Perhaps this might be the reason why the second-line nurses (Median = 7, [IQR 5–9]) were more worried about their health than the front-line nurses (Median = 6, [IQR 5–8]). Compared to the front-line nurses, the second-line nurses thought their departments unprepared for the pandemic, a perception that might be related to the shortage of protective equipment (32). Because avoiding patient contact and wearing personal protective equipment are the most effective ways to reduce the risk of infection (33, 34). Eighty-eight percent of the nurses thought the epidemic was dangerous. This proportion was much higher than 61% of the nurses worried about the H1N1 pandemic (35). This might have something to do with the lack of clarity about the diagnosis and treatment of pneumonia (36). Second-line nurses thought COVID-19 was harder to treat than did the front-line nurses, and more second-line nurses (36%) thought that the epidemic would affect their careers more than did the front-line nurses (26%). This was related to the fact that front-line nurses took direct care of the diagnosed patents. Thus, they had direct access to information on diagnosis and treatment of COVID-19. At the second line, if a patient was suspected to be infected, she/he would be transferred to the front line. They had no contact with those confirmed to have COVID-19; however, they found it difficult to identify infected patients from the general patient population. In general, the second-line nurses were in more distress than we thought. Both front- and second-line nurses want more health information. There was no difference in the perception for symptoms, prognosis, and transmission of COVID-19 between the front-line nurses and the second-line nurses. This may be because the National Health Commission of the People's Republic of China requires all departments to share relevant data (37). The front-line nurses knew more about the treatment of COVID-19 than did the second-line nurses because they were caring for these patients. And they were informed more about their health than were second-line nurses. But the second-line nurses thought that they knew more about the prevention of COVID-19 than did the front-line nurses. During the outbreak, China strengthened online medical services and telephone follow-up and arranged orderly treatment for non-emergency patients (38). For fear of infection, some people avoided hospitals as much as possible. Some second-line nurses said that they cared for fewer patients during the outbreak, so they spent time to learn more about prevention. They can communicate and share information on the Internet and over the phone, so the second-line nurses can get a lot of information about COVID-19. Therefore, how to share the latest information about the epidemic quickly needs to be addressed in future outbreaks of infectious diseases. The media may be a good choice. Current research suggests that media-induced fear regulation could be used as an important non-pharmaceutical intervention to alleviate the pandemic. And media influence plays an important role in the dissemination of useful information in a variety of ways (39). During the outbreak, almost all of the nurses volunteered to go to the front line to fight the outbreak. Very few nurses (1.5%) thought that they might take time off out of concern for the infection. Most nurses thought their working conditions were dangerous, and 77% limited their social contacts, as did medical workers during the 2003 SARS outbreak (40), and this percentage was much higher than 7% who limited their social contacts during the 2009 influenza virus and A/H1N1 outbreaks (35). In the COVID-19 emergency, nurses had little inclination to evade their duties. Front-line nurses were less likely to avoid their responsibilities than were second-line nurses. About one-third of nurses believed that family and friends avoided contact with them, and front-line nurses reported this avoidance more than did second-line nurses, possibly because they knew they were directly exposed to the virus. This distancing confirms the results of another study that showed spatial and social distance were important predictors of public attention to pandemics (41). The government and communities also restricted frequent visits and large gatherings to prevent the spread of the virus, which also limited the nurse's socialization and contact with family and friends. At the same time, the front-line nurses received more support (42). Especially in terms of “social gratitude,” “hospital protection and arrangements of temporary accommodation,” “whether to provide insurance and compensation when infected in the workplace,” “new work arrangements and clear guidelines for infection control,” “receiving front-line works' feedback by administrative staff,” and “psychological counseling for employees organized by superior management departments or hospitals.” But there was no difference between front- and second-line nurses in “Support from relatives,” “Sense of coherence and team spirit,” “Gratitude from patients and their relatives,” “Expressing opinions through staff unions or mass media.” The front-line nurses got psychological intervention, including face-to-face, over the phone, or online. But we did not find one psychological survey about nurses involved in COVID-19, so we didn't know what evidence these interventions were based on. It was impossible to judge whether these interventions were beneficial to nurses. Medical workers experienced significant stress during infectious epidemics. We found that 28% of nurses reported mild-to-moderate distress and 6% reported serious distress. The proportion of nurses reporting mild-to-moderate stress (24%) was higher than that of nurses during A/H1N1 influenza pandemic. However, this proportion of nurses with severe distress was lower than that of the general hospital staff during the A/H1N1 influenza pandemic (9%) (35). The difference may be explained by the fact that this study was conducted after the A/H1N1 outbreak, whereas ours was conducted during the COVID-19 outbreak. Some of the nurses said that their main focus was on treating patients and had little time to think about other things. Researchers found the opposite in a study in Singapore among medical workers during the SARS outbreak. Whereas 30% of front-line nurses reported mild-to-moderate distress, 26% of second-line nurses reported mild-to-moderate distress (5). This difference may be explained by the higher number of infected patients and the larger size of the affected areas of the COVID-19 outbreak. Distress was mild-to-moderate in 28% of all nurses and severe in 6%. Second-line nurses reported more distress than did first-line nurses. Our analysis showed nurses who were unmarried or divorced, lived alone, and had higher support scores were less worried about the outbreak. So more attention should be paid to the nurses' concerns about a pandemic, who get married or live with their family. Every one-point increase in the total support score reduced the risk of distress by about 25%. Therefore, more support should be given to both front- and second-line nurses to reduce their distress. Some front-line nurses said they paid more attention to the patients than themselves, so we inferred that treating infected patients maybe was protective against distress. After the outbreak is over, the front-line nurses may be at increased risk for distress. Therefore, when the outbreak is over, they may need early intervention to prevent and treat anxiety.

### Limitations of the Study

The greatest limitation to our study was the use of snowball sampling. However, although we cannot say that the nurses who responded are a representative sample, the nurses who did respond provided clear evidence of distress and concerns, as well a perceived lack of information and social support. Another limitation but also a strength of the survey was that it was conducted during the COVID-19 outbreak. Our response rate was almost certainly affected by the fatigue and stress that accompanied continuous intensive work, and because the nurses were self-selecting, we cannot rule out response bias. We also had no baseline data against which to compare the outbreak.

## Conclusion

During the COVID-19 epidemic, the nurses involved were under great psychological pressure and the second-line nurses were more stressed than the front-line nurses. Nurses who lived alone and felt supported had lower levels of anxiety. Nurses should be screened for psychological problems as part of the emergency epidemic prevention and control system, and appropriate interventions should be implemented as soon as possible during the epidemic.

## Data Availability Statement

The original contributions presented in the study are included in the article/supplementary materials, further inquiries can be directed to the corresponding author/s.

## Ethics Statement

The studies involving human participants were reviewed and approved by Biomedical Research Ethics Committee, West China Hospital of Sichuan University. The patients/participants provided their written informed consent to participate in this study.

## Author Contributions

JH, LD, and GL designed the study. YLiu, LYe, KT, XA, FZ, XS, and CS recruited the respondents. LD, YLon, QG, YCh, YLin, and LYa collected and analyzed the data. YLiu, YLon, YCh, QG, and LYa drafted the manuscript. LD, JH, CS, YLin, YCa, YJ, and KL undertook a critical revision of the manuscript. All authors contributed to the article and approved the submitted version.

## Conflict of Interest

The authors declare that the research was conducted in the absence of any commercial or financial relationships that could be construed as a potential conflict of interest.

## References

[1] Wang FS Zhang C. What to do next to control the 2019-nCoV epidemic? Lancet. (2020) 395:391–93. 10.1016/S0140-6736(20)30300-7 32035533 PMC7138017

[2] Xiang YT Li W Zhang Q Jin Y Rao WW Zeng LN. Timely research papers about COVID-19 in China. Lancet. (2020) 395:684–5. 10.1016/S0140-6736(20)30375-5 32078803 PMC7133590

[3] Khajanchi S Sarkar K. Forecasting the daily and cumulative number of cases for the COVID-19 pandemic in India. Chaos. (2020) 30:071101. 10.1063/5.0016240 32752627 PMC7585452

[4] Chen WK Cheng YC Chung YT Lin CC. The impact of the SARS outbreak on an urban emergency department in Taiwan. Med Care. (2005) 43:168–72. 10.1097/00005650-200502000-00010 15655430

[5] Chan AO Huak CY. Psychological impact of the 2003 severe acute respiratory syndrome outbreak on health care workers in a medium size regional general hospital in Singapore. Occup Med (Lond). (2004) 54:190–6. 10.1093/occmed/kqh027 15133143 PMC7107861

[6] Khalid I Khalid TJ Qabajah MR Barnard AG Qushmaq IA. Healthcare workers, emotions, perceived stressors and coping strategies during a MERS-CoV outbreak. Clin Med Res. (2016) 14:7–14. 10.3121/cmr.2016.1303 26847480 PMC4851451

[7] Akdeniz G Kavakci M Gozugok M Yalcinkaya S Kucukay A Sahutogullari B. A survey of attitudes, anxiety status, and protective behaviors of the university students during the COVID-19 outbreak in Turkey. Front Psychiatry. (2020) 11:695. 10.3389/fpsyt.2020.00695 32760303 PMC7373786

[8] Wang W Song W Xia Z He Y Tang L Hou J. Sleep disturbance and psychological profiles of medical staff and non-medical staff during the early outbreak of COVID-19 in Hubei Province, China. Front Psychiatry. (2020) 11:733. 10.3389/fpsyt.2020.00733 32793014 PMC7387679

[9] Khee KS Lee LB Chai OT Loong CK Ming CW Kheng TH The psychological impact of SARS on health care providers. Crit Care Shock. (2004) 7:99–106.

[10] Maunder R Hunter J Vincent L Bennett J Peladeau N Leszcz M. The immediate psychological and occupational impact of the 2003. SARS outbreak in a teaching hospital. CMAJ. (2003) 168:1245–51. 12743065 PMC154178

[11] Bai Y Lin CC Lin CY Chen JY Chue CM Chou P. Survey of stress reactions among health care workers involved with the SARS outbreak. Psychiatr Serv. (2004) 55:1055–7. 10.1176/appi.ps.55.9.1055 15345768

[12] Koh D Lim MK Chia SE Ko SM Qian F Ng V. Risk perception and impact of Severe Acute Respiratory Syndrome (SARS) on work and personal lives of healthcare workers in Singapore: what can we learn? Med Care. (2005) 43:676–82. 10.1097/01.mlr.0000167181.36730.cc 15970782

[13] Wong TW Yau JK Chan CL Kwong RS Ho SM Lau CC. The psychological impact of severe acute respiratory syndrome outbreak on healthcare workers in emergency departments and how they cope. Eur J Emerg Med. (2005) 12:13–8. 10.1097/00063110-200502000-00005 15674079

[14] Vagni M Maiorano T Giostra V Pajardi D. Coping with COVID-19: emergency stress, secondary trauma and self-efficacy in healthcare and emergency workers in Italy. Front Psychol. (2020) 11:566912. 10.3389/fpsyg.2020.566912 33013603 PMC7494735

[15] Zhang WR Wang K Yin L Zhao WF Xue Q Peng M. Mental health and psychosocial problems of medical health workers during the COVID-19 epidemic in China. Psychother Psychosom. (2020) 89:242–50. 10.1159/000507639 32272480 PMC7206349

[16] García-Fernández L Romero-Ferreiro V López-Roldán PD Padilla S Calero-Sierra I Monzó-García M. Mental health impact of COVID-19 pandemic on Spanish healthcare workers. Psychol Med. (2020). 10.1017/S0033291720002019. [Epub ahead of print]. 32456735 PMC7272696

[17] Daugherty AM Arble EP. Prevalence of mental health symptoms in residential healthcare workers in Michigan during the COVID-19 pandemic. Psychiatry Res. (2020) 291:113266. 10.1016/j.psychres.2020.113266 32623265 PMC7324917

[18] “News 1+1” | Yansong B dialogued with Xinjuan W chairman of Chinese Nursing Association and Peihong W (The Head Nurse of Jianghan Square Cabin Hospital) Special topic. Chin J Mod Nurs. (2020). Available online at: http://www.cjmn.net/article/content/view?id=7898 (in Chinese).

[19] Samui P Mondal J Khajanchi S. A mathematical model for COVID-19 transmission dynamics with a case study of India. Chaos Solitons Fractals. (2020) 140:110173. 10.1016/j.chaos.2020.110173 32834653 PMC7405793

[20] Vieweg Bw Hedlund JL The General Health Questionnaire (GHQ): a comprehensive review. J Oper Psychiatr. (1983) 14:74–81.

[21] Garyfallos G Karastergiou A Adamopoulou A Moutzoukis C Alagiozidou E Mala D., Garyfallos A: Greek version of the General Health Questionnaire: accuracy of translation and validity. Acta Psychiatr Scand. (1991). 84:371–8. 10.1111/j.1600-0447.1991.tb03162.x 1746290

[22] Sarkar K Khajanchi S Nieto JJ. Modeling and forecasting the COVID-19 pandemic in India. Chaos Solitons Fractals. (2020) 139:110049. 10.1016/j.chaos.2020.110049 32834603 PMC7321056

[23] Olu O Kargbo B Kamara S Wurie AH Amone J Ganda L. Epidemiology of Ebola virus disease transmission among health care workers in Sierra Leone, May to December 2014: a retrospective descriptive study. BMC Infect Dis. (2015) 15:416. 10.1186/s12879-015-1166-7 26464285 PMC4604711

[24] Wise ME DePerio M Halpin J Jhung M Magill S Black SR. Transmission of pandemic (H1N1) 2009 influenza to healthcare personnel in the United States. Clin Infect Dis. (2011) 52 Suppl 1:S198–204. 10.1093/cid/ciq038 21342895

[25] Kang L Li Y Hu S Chen M Yang C Yang BX. The mental health of medical workers in Wuhan, China dealing with the 2019 novel coronavirus. Lancet. (2020) 7:e14. 10.1016/S2215-0366(20)30047-X 32035030 PMC7129673

[26] NEWS 1+1 Today's Outbreak Response: Nurses on the Front Line. Available online at: http://tv.cctv.com/2020/02/11/VIDEuntxUaAVJtc9ZDkUfkzp200211.shtml (in Chinese).

[27] Liu C Wang H Zhou L Xie H Yang H Yu Y. Sources and symptoms of stress among nurses in the first Chinese anti-Ebola medical team during the Sierra Leone aid mission: a qualitative study. Int J Nurs Sci. (2019) 6:187–91. 10.1016/j.ijnss.2019.03.007 31406890 PMC6608674

[28] Li Q Guan X Wu P Wang X Zhou L Tong Y. Early transmission dynamics in Wuhan, China, of novel coronavirus-infected pneumonia. N Engl J Med. (2020) 382:1199–207. 10.1056/NEJMoa2001316 31995857 PMC7121484

[29] Huang C Wang Y Li X Ren L Zhao J Hu Y. Clinical features of patients infected with 2019 novel coronavirus in Wuhan, China. Lancet. (2020) 395:497–506. 10.1016/S0140-6736(20)30183-5 31986264 PMC7159299

[30] Li Y Wang H Jin XR Li X Pender M Song CP. Experiences and challenges in the health protection of medical teams in the Chinese Ebola treatment center, Liberia: a qualitative study. Infect Dis Poverty. (2018) 7:92. 10.1186/s40249-018-0468-6 30134982 PMC6103862

[31] Ngatu NR Kayembe NJ Phillips EK Okech-Ojony J Patou-Musumari M Gaspard-Kibukusa M. Epidemiology of ebolavirus disease (EVD) and occupational EVD in health care workers in Sub-Saharan Africa: need for strengthened public health preparedness. J Epidemiol. (2017) 27:455–61. 10.1016/j.je.2016.09.010 28416172 PMC5602796

[32] National Health Commission of the People's Republic of China Transcript of Press Conference (2020). Available online at: http://www.nhc.gov.cn/xcs/fkdt/202002/401d6ca349014931bd5d993ed0e4519e.shtml (accessed February 23, 2020) (in Chinese).

[33] Koh Y Hegney DG Drury V. Comprehensive systematic review of healthcare workers' perceptions of risk and use of coping strategies towards emerging respiratory infections diseases. Int J Evid Based Health. (2011) 9:403–19. 10.1111/j.1744-1609.2011.00242.x 22093389

[34] Yiwen K Hegney D Drury V. A comprehensive systematic review of healthcare workers' perceptions of risk from exposure to emerging acute respiratory infectious diseases and the perceived effectiveness of strategies used to facilitate healthy coping in acute hospital and community healthcare settings. JBI Libr Syst Rev. (2010) 8:917–71. 10.11124/jbisrir-2010-150 27819952

[35] Goulia P Mantas C Dimitroula D Mantis D Hyphantis T. General hospital staff worries, perceived sufficiency of information and associated psychological distress during the A/H1N1 influenza pandemic. BMC Infect Dis. (2010) 10:322. 10.1186/1471-2334-10-322 21062471 PMC2990753

[36] Ying-Hui J Lin C Zhen-Shun C Cheng H Deng T Fan YP A rapid advice guideline for the diagnosis and treatment of 2019 novel coronavirus (2019-nCoV) infected pneumonia (standard version). Mil Med Res. (2020) 7:4 10.1186/s40779-020-0233-6 32029004 PMC7003341

[37] National Health Commission of the People's Republic of China Notice on the Prevention and Control of Pneumonia Epidemic Caused by New Coronavirus for Reducing the Burden on the Community. Available online at: http://www.nhc.gov.cn/xcs/zhengcwj/202002/b2b06414c0f44e1db28d56e11044ba3b.shtml (accessed February 23,2020) (in Chinese).

[38] National Health Commission of the People's Republic of China Notice of the General Office of the National Health Commission on Strengthening Medical Service Management to Meet the Basic Medical Needs of the Masses During the Epidemic. Available online at: http://www.nhc.gov.cn/yzygj/s7659/202002/6d5a8556c5ce46368263711698d8237a.shtml. (accessed February 17,2020) (in Chinese).

[39] Kumar S Sharma B Singh V Modelling the role of media induced fear conditioning in mitigating post-lockdown COVID-19 pandemic: perspectives on India. arXiv:2004.13777. (2020).

[40] Tam CW Pang EP Lam LC Chiu HF. Severe acute respiratory syndrome (SARS) in Hong Kong in 2003: stress and psychological impact among frontline healthcare workers. Psychol Med. (2004) 34:1197–204. 10.1017/S0033291704002247 15697046

[41] van Lent LGG Sungur H Kunneman FA van de Velde B Das E. Too far to care? Measuring public attention and fear for ebola using Twitter. J Med Internet Res. (2017) 19:e193. 10.2196/jmir.7219 28611015 PMC5487741

[42] National Health Commission of the People's Republic of China Notice on the Issuance of COVID-19 Epidemic Psychological Counseling Work Program. Available online at: http://www.nhc.gov.cn/jkj/s3577/202003/0beb22634f8a4a48aecf405c289fc25e.shtml (accessed March 18,2020) (in Chinese).

